# Racial disparities in the relationship of regional socioeconomic status and colorectal cancer survival in the five regions of Georgia

**DOI:** 10.1002/cam4.6954

**Published:** 2024-02-13

**Authors:** Meng‐Han Tsai, Marlo Vernon, Shaoyong Su, Steven S. Coughlin, Yanbin Dong

**Affiliations:** ^1^ Cancer Prevention, Control & Population Health Program, Georgia Cancer Center Augusta University Augusta Georgia USA; ^2^ Georgia Prevention Institute, Augusta University Augusta Georgia USA; ^3^ Department of Biostatistics, Data Science and Epidemiology Augusta University Augusta Georgia USA

**Keywords:** colorectal cancer, Georgia, racial disparities, regional socioeconomic status

## Abstract

**Introduction:**

The study's purpose was to examine 5‐year colorectal cancer (CRC) survival rates between White and Black patients. We also determined whether regional socioeconomic status (SES) is associated with CRC survival between White and Black patients in the Clayton, West Central, East Central, Southeast, and Northeast Georgia public health districts.

**Methods:**

We performed a retrospective cohort analysis using data from the 1975 to 2016 Surveillance, Epidemiology, and End Results program. The 2015 United States Department of Agriculture Economic Research Services county typology codes were used to identify region‐level SES with persistent poverty, low employment, and low education. Kaplan–Meier method and Cox proportional hazard regression were performed.

**Results:**

Among 10,876 CRC patients (31.1% Black patients), 5‐year CRC survival rates were lower among Black patients compared to White patients (65.4% vs. 69.9%; *p* < 0.001). In multivariable analysis, White patients living in regions with persistent poverty had a 1.1‐fold increased risk of CRC death (HR, 1.12; 95% CI, 1.00–1.25) compared to those living in non‐persistent poverty regions. Among Black patients, those living in regions with low education were at a 1.2‐fold increased risk of CRC death (HR, 1.19; 95% CI, 1.01–1.40) compared to those living in non‐low education regions.

**Discussion and Conclusions:**

Black patients demonstrated lower CRC survival rates in Georgia compared to their White counterparts. White patients living in regions with persistent poverty, and Black patients living in regions with low education had an increased risk of CRC death. Our findings provide important evidence to all relevant stakeholders in allocating health resources aimed at CRC early detection and prevention and timely referral for CRC treatment by considering the patient's regional SES in Georgia.

## 1 INTRODUCTION

Colorectal cancer (CRC) is the primary cause of cancer death in Georgia,^1^ with a significantly higher burden of CRC mortality found in the Clayton, East Central, West Central, Northeast, and Southeast public health districts of Georgia in comparison to Georgia as a whole during 2008–2013.^1^ Several Georgia counties within these districts, such as Clayton, Richmond (East Central region), and Muscogee (West Central region), could be considered as lower socioeconomic areas (SES) with 19%–21% of residents living in poverty in comparison to overall proportions in Georgia.^2^ CRC mortality rates vary considerably by race/ethnicity in Georgia,^3^, ^4^ with non‐Hispanic Black (hereafter Black) having the highest mortality compared to non‐Hispanic White (hereafter White),^4^ even though the availability of screening and treatment options for CRC have improved for both race groups. Differences in SES at the individual level (e.g., education, occupation, or income),^5^, ^6^ are intimately tied to area‐level SES,^7^, ^8^ and may explain racial disparities in CRC outcomes.

Low area‐level SES has been linked to detrimental cancer outcomes in many studies, including neighborhood‐level measures of low education, low employment, and poverty.^9^, ^10^ Evidence has shown that cancer outcomes are often poorer for patients living in low SES neighborhoods with a majority of racial minorities (e.g., Black).^8^, ^11^ For example, Black patients living in neighborhoods with lower income, education, and employment have been linked to worse survival among those with non‐metastatic CRC.^8^ Patients of any race living in socioeconomically disadvantaged neighborhoods also have a 15%–25% increased risk of death due to CRC.^12^, ^13^ Barriers associated with poorer outcomes, including limited availability of cancer screening and treatment options as well as transportation barriers, exist among patients of low SES communities.^14^, ^15^ Therefore, identifying regional SES may significantly contribute to improving outcomes through the allocation of medical resources (e.g., CRC screening and treatment options) for underserved populations and their communities in Georgia.

Although a few studies examining racial disparities in CRC survival considered neighborhood SES, they primarily focused on large geographic areas by using a composite index.^16^, ^17^ The influence of specific SES at a regional level on CRC survival in smaller geographic areas is not well understood, particularly the comparison of different racial groups. A study of CRC survival profiles considering area‐level SES within small areas is essential to inform local policies for allocating appropriate health resources. To address the gaps, we sought to (1) examine the 5‐year CRC survival rates between White and Black patients and (2) determine whether patients living in low SES regions, including counties with “persistent poverty,” “low employment,” and “low education”—as provided by the US Department of Agriculture Economic Research Services (USDA ERS) and, are at greater risk of CRC death in the Clayton, East Central, West Central, Northeast, and Southeast regions of Georgia.

## 2 METHODS

### 2.1 Study design

Data from the Surveillance, Epidemiology, and End Results (SEER) Program were used, which is a source for comprehensive population‐based information in the US that includes patient demographics, primary tumor site, tumor morphology and stage at diagnosis, first course of treatment, and follow‐up for vital status. The study‐eligible population included patients diagnosed with CRC defined by the SEER Site Recode ICD‐O‐3/WHO 2008 definition of colon cancer (C180–C189), rectosigmoid junction cancer (C199), and rectal cancer (C209).^18^ In addition, we used the county Federal Information Processing System (FIPS) code for the State of Georgia “13” and county for coding five regions of Georgia with significantly higher CRC mortality rates,^19^, ^20^ including one county in Clayton, 13 counties in the East Central, 16 counties in the West Central, 22 counties in the Southeast, and 10 counties in the Northeast regions.^1^ These five regions of Georgia were defined by Georgia public health districts.^1^ Data extracted for this study were publicly available and de‐identified, and thus considered exempt from Institutional Review Board (IRB) review.

### 2.2 Study participants

A total of 997,685 CRC patients were included in SEER for 1975–2016. To obtain an eligible study sample, we excluded 247,033 CRC patients aged under 18 years (*n* = 675), repeated diagnosis of CRC (*n* = 44,809), missing rural and urban information (*n* = 14,109), CRC diagnosed after 2011 due to limited follow‐up time (<5 years) (*n* = 177,707), missing survival time (*n* = 9681), and missing cancer sites (*n* = 52). Further, we excluded 739,629 CRC patients who did not live in Georgia (*n* = 689,736) or the five regions of Georgia (*n* = 49,893). Finally, we excluded 147 patients who were “other race” (e.g., Hispanic) due to a low representative sample. As a result, 10,876 CRC patients living within five public health districts of Georgia were included, with 7507 White patients and 3369 Black patients in the final sample for statistical analysis.

### 2.3 Measures

CRC survival was the outcome of interest and regional SES was the primary exposure of interest. For regional SES, counties with “persistent poverty, low employment, and low education” were identified through the 2015 USDA ERS county typology codes.^21^, ^22^ A county is classified by the USDA ERS as a “persistent poverty” county if more than 20% of residents were considered poor by the 1980–2000 decennial census, and the American Community 5‐year estimates for 2007–2011. The USDA ERS designates a “low employment” county if fewer than 65% of residents aged 25–64 were employed in 2008–2012, and as a “low education” county if more than 20% of residents did not have a high school diploma or equivalent during the same period. We linked regional SES by using county FIPS code as identifiers to link cancer registries and SES database for CRC patients in these five regions of Georgia. Finally, persistent poverty, low employment, and low education were classified as three two‐level variables: (1) yes or (2) no.

Moreover, individual‐level covariates included patient demographics and tumor features were adjusted for their impact on the relationship between regional SES and CRC survival. Inpatient demographics included age at diagnosis (18–49, 50–64, or ≥65), gender (male or female), marital status (single, married, others, or unknown), and Georgia regional residence (Clayton, West Central, East Central, Southeast, or Northeast regions). In tumor features, we included grade (Grades 1, 2, 3 and 4, or unknown), stage at diagnosis (localized, regionalized, distant, or unknown), and primary site (right or left).

### 2.4 Statistical analysis

Descriptive statistics were used to describe the distribution of CRC patients by race (White and Black), regional SES, patient demographics, and tumor features. We examined bivariate differences across different racial groups in regional SES, patient demographics, and tumor features, using the chi‐square test. Survival analyses at 5‐year intervals were applied using the Kaplan–Meier (KM) method. The Log‐Rank test was performed to compare the survival rates within White and Black patients and stratified by persistent poverty, low employment, and low education areas. Further, we performed Cox proportional hazard regression to examine the impact of regional SES on CRC survival among White and Black patients. Three sequential regression models were performed to examine the association between White and Black patients, respectively. Model 1 only included regional SES; Model 2 was further adjusted for patient demographics; and Model 3 was further adjusted for tumor features. The county designations of low employment were removed from all regression models due to a lack of statistically significant difference. Such survival analyses methods (KM curves with log‐rank test and Cox proportional hazard regression) enable censoring patients to the specific events (patient death due to CRC) by the end of the study period. Therefore, we measured CRC patients' survival time in months from the date of diagnosis up to 60 months of follow‐up, censored at the end of the study observation period (December 31, 2016), or death due to CRC. We conducted analyses using SAS Version 9.4, SAS Institute Inc., Cary, North Carolina. All results were reported using hazard ratios (HRs) and the associated 95% confidence intervals (CIs). The level of statistical significance was set at an alpha level of 0.05 and the *p*‐values were based on two‐sided probability tests.

## 3 RESULTS

### 3.1 Patient characteristics

Table 1 describes the characteristics of regional SES, patient demographics, tumor features, and stratified by race groups. In these five regions of Georgia, a majority of CRC patients lived in non‐low education (77.1%), non‐low employment (60.9%), and non‐persistent poverty (72.2%) areas. Inpatient demographics and tumor features, we observed that most CRC patients were aged 65 years or older, male, married, East Central residents, had localized CRC, had Grade 2 disease, and CRC found in the left colon. Similarly, among White and Black patients, a majority live in non‐low employment and non‐persistent poverty areas (all *p* < 0.001). When exploring the racial differences, we found that more Black patients lived in low employment (44%) and persistent poverty (31.1%) areas compared to White patients living in these mentioned areas (36.9% and 26.4%), respectively (*p* < 0.001). Higher rates of young CRC diagnosis (18–49 years) were found among Black patients (15.7%) compared to White patients in the same age group (10.1%) (*p* < 0.001). Importantly, Black patients had more late‐stage diagnoses of CRC compared to White patients (22.8% vs. 18.3%; *p* < 0.001).

**TABLE 1:** Regional socioeconomic status, demographics, and tumor features of colorectal cancer patients stratified by race groups (*n* = 10,876).

	Total (*n* = 10,876)	White (*n* = 7507)	Black (*n* = 3379)	*p*‐value
	*n* (%)	*n* (%)	*n* (%)	
*Regional socioeconomic status*				
Low education				
No	8381 (77.1%)	5818 (77.5%)	2563 (76.1%)	0.102
Yes	2495 (22.9%)	1689 (22.5%)	806 (23.9%)	
Low employment				
No	6625 (60.9%)	4739 (63.1%)	1886 (56.0%)	<0.001
Yes	4251 (39.1%)	2768 (36.9%)	1483 (44.0%)	
Persistent poverty				
No	7849 (72.2%)	5529 (73.7%)	2320 (68.9%)	<0.001
Yes	3027 (27.8%)	1978 (26.4%)	1049 (31.1%)	
*Demographics*				
Age at diagnosis				
18–49 years	1284 (11.8%)	756 (10.1%)	528 (15.7%)	<0.001
50–64 years	3784 (34.8%)	2421 (32.3%)	1363 (40.5%)	
≥65 years	5808 (53.4%)	4330 (57.7%)	1478 (43.9%)	
Gender				
Female	5202 (47.8%)	3476 (46.3%)	1726 (51.2%)	<0.001
Male	5674 (52.2%)	4031 (53.7%)	1643 (48.8%)	
Marital status				
Single	1331 (12.2%)	603 (8.0%)	728 (21.6%)	<0.001
Married	5875 (54.0%)	4489 (59.8%)	1386 (41.1%)	
Others^a^	3272 (30.1%)	2183 (29.1%)	1089 (32.3%)	
Unknown	398 (3.7%)	232 (3.1%)	166 (4.9%)	
Georgia regional residence				
Clayton	1955 (18.0%)	1336 (17.8%)	619 (18.4%)	<0.001
West Central	2009 (18.5%)	1200 (16.0%)	809 (24.0%)	
East Central	2542 (23.4%)	1520 (20.3%)	1022 (30.3%)	
Southeast	2138 (19.7%)	1673 (22.3%)	465 (13.8%)	
Northeast	2232 (20.5%)	1778 (23.7%)	454 (13.5%)	
*Tumor features*				
Stage				
Localized	4172 (38.4%)	2907 (38.7%)	1265 (37.6%)	<0.001
Regionalized	4002 (36.8%)	2869 (38.2%)	1133 (33.6%)	
Distant	2142 (19.7%)	1374 (18.3%)	768 (22.8%)	
Unknown	560 (5.2%)	357 (4.8%)	203 (6.0%)	
Grade^b^				
Grade 1	1213 (11.2%)	843 (11.2%)	370 (11.0%)	<0.001
Grade 2	6516 (59.9%)	4515 (60.1%)	2001 (59.4%)	
Grades 3 and 4	1421 (13.1%)	1080 (14.4%)	341 (10.1%)	
Unknown	1726 (15.9%)	1069 (14.2%)	657 (19.5%)	
Primary site				
Right	4251 (39.1%)	2887 (38.5%)	1364 (40.5%)	0.045
Left	6625 (60.9%)	4620 (61.5%)	2005 (59.5%)	

^a^ Others include divorced, separated, and widow.

^b^ Grade 1: well‐differentiated; Grade 2: moderately differentiated; Grade 3: poorly differentiated; Grade 4: undifferentiated.

### 3.2 Five‐year CRC survival rates

**FIGURE 1:**
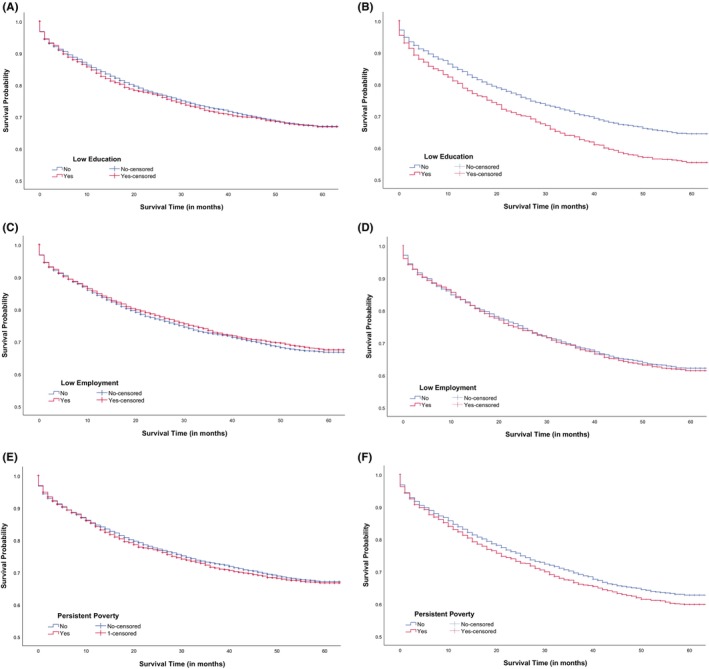
Kaplan–Meier colorectal cancer survival curves by race groups and regional socioeconomic status. Log‐rank test was performed to examine survival rates.

The average survival time since CRC diagnosis for White patients was 74.3 months (SD, 68.3 months), and for Black patients was 65.6 months (SD, 58.1 months). The 5‐year CRC survival rates were lower among Black patients compared to White patients (65.4% vs. 69.9%; *p* < 0.001). When examining survival rates in Black patients within different regional SES, those living in low‐education (59.4% vs. 67.3%, *p* < 0.001; Figure 1B), low employment (65.4% vs. 65.3%, *p* = 0.674; Figure 1D), and persistent poverty (64.0% vs. 66.0%, *p* = 0.115; Figure 1F) regions appeared to report lower 5‐year survival rates compared to those not living in those regions, respectively. Significant differences in 5‐year survival rates were not found among White patients in low education (Figure 1A), low employment (Figure 1C), and persistent poverty (Figure 1E **)** regions.

### 3.3 Regional SES on CRC survival

Results from three sequential models were slightly different among White patients (Table 2). In the full model, White patients living in persistent poverty areas had a 12% higher risk of CRC death compared to those not living in persistent poverty (HR, 1.12; 95% CI, 1.00–1.25). In Table 3, among Black patients, those living in low‐education areas demonstrated a greater CRC risk of death compared to those not living in these areas (HR, 1.19; 95% CI, 1.01–1.40). Similar results were found in two reduced models with a 1.4‐fold increased risk of CRC death for Black patients living in low‐education areas. Other factors associated with increased risk of CRC death include older age at diagnosis (≥65 years), distant stage of diagnosis, and Grades 3 and 4 disease regardless of race groups (*p* < 0.001) (Tables 2 and 3).

**TABLE 2:** Association between regional socioeconomic status and CRC survival among White patients.

	Model 1^c^		Model 2^c^		Model 3^c^	
	HR (95% CI)	*p*‐value	HR (95% CI)	*p*‐value	HR (95% CI)	*p*‐value
*Regional socioeconomic status*						
Low education						
No	Reference	0.859	Reference	0.429	Reference	0.364
Yes	1.01 (0.91, 1.12)		1.05 (0.94, 1.17)		1.05 (0.94, 1.18)	
Persistent poverty						
No	Reference	0.746	Reference	0.359	Reference	0.043
Yes	1.02 (0.92, 1.13)		1.05 (0.95, 1.17)		*1.12 (1.00, 1.25)*	
*Demographics*						
Age at diagnosis						
18–49	NA		Reference	<0.001	Reference	<0.001
50–64	NA		0.90 (0.77, 1.05)		1.09 (0.93, 1.27)	
≥65 years	NA		*1.26 (1.09, 1.46)*		*1.80 (1.55, 2.08)*	
Gender						
Male	NA		Reference	<0.001	Reference	<0.001
Female	NA		*0.81 (0.74, 0.89)*		*0.82 (0.75, 0.90)*	
Marital status						
Single	NA		Reference	<0.001	Reference	<0.001
Married	NA		*0.63 (0.55, 0.73)*		*0.73 (0.63, 0.85)*	
Others^a^	NA		0.96 (0.83, 1.13)		0.94 (0.80, 1.09)	
Unknown	NA		*0.72 (0.55, 0.96)*		0.90 (0.68, 1.20)	
Georgia regional residence						
West Central	NA		Reference	<0.001	Reference	<0.001
Clayton	NA		*1.28 (1.11, 1.48)*		*1.33 (1.15, 1.54)*	
East Central	NA		1.08 (0.94, 1.24)		1.08 (0.93, 1.24)	
Northeast	NA		1.00 (0.87, 1.14)		0.96 (0.84, 1.11)	
Southeast	NA		0.99 (0.86, 1.14)		1.00 (0.87, 1.15)	
*Tumor features*						
Stage						
Localized	NA		NA		Reference	<0.001
Regionalized	NA		NA		*3.45 (2.99, 3.98)*	
Distant	NA		NA		*19.86 (17.26, 22.85)*	
Unknown	NA		NA		*5.87 (4.78, 7.20)*	
Grade^b^						
Grade 1	NA		NA		Reference	<0.001
Grade 2	NA		NA		*1.22 (1.03, 1.45)*	
Grades 3 and 4	NA		NA		*2.11 (1.75, 2.53)*	
Unknown	NA		NA		*2.03 (1.67, 2.45)*	
Primary site						
Left	NA		NA		Reference	0.481
Right	NA		NA		0.94 (0.86, 1.02)	

*Note*: Italicized text indicates statistically significant result.

Abbreviations: CRC, colorectal cancer; HR, hazard ratio.

^a^ Others include divorced, separated, and widow.

^b^ Grade 1: well‐differentiated; Grade 2: moderately differentiated; Grade 3: poorly differentiated; Grade 4: undifferentiated.

^c^ Model 1 was adjusted for regional socioeconomic status; model 2 was adjusted for regional socioeconomic status and demographics; model 3 was adjusted for regional socioeconomic status, demographics, and tumor features.

**TABLE 3:** Association between regional socioeconomic status and CRC survival among Black patients.

	Model 1^c^		Model 2^c^		Model 3^c^	
	HR (95% CI)	*p*‐value	HR (95% CI)	*p*‐value	HR (95% CI)	*p*‐value
*Regional socioeconomic status*						
Low education						
No	Reference	<0.001	Reference	<0.001	Reference	0.040
Yes	*1.37 (1.18, 1.60)*		*1.40 (1.19, 1.64)*		*1.19 (1.01, 1.40)*	
Persistent poverty						
No	Reference	0.400	Reference	0.305	Reference	0.795
Yes	0.94 (0.81, 1.09)		0.92 (0.79, 1.08)		0.98 (0.84, 1.15)	
*Demographics*						
Age at diagnosis						
18–49	NA		Reference	0.001	Reference	<0.001
50–64	NA		0.87 (0.74, 1.03)		0.97 (0.81, 1.14)	
≥65 years	NA		1.13 (0.95, 1.34)		*1.47 (1.24, 1.75)*	
Gender						
Male	NA		Reference	<0.001	Reference	0.060
Female	NA		*0.80 (0.71, 0.90)*		0.89 (0.79, 1.01)	
Marital status						
Single	NA		Reference	<0.001	Reference	<0.001
Married	NA		*0.69 (0.59, 0.80)*		*0.79 (0.68, 0.92)*	
Others^a^	NA		0.96 (0.82, 1.13)		1.06 (0.90, 1.24)	
Unknown	NA		0.74 (0.54, 1.00)		0.90 (0.66, 1.23)	
Georgia regional residence						
West Central	NA		Reference	0.147	Reference	0.377
Clayton	NA		1.06 (0.88, 1.27)		0.98 (0.81, 1.18)	
East Central	NA		0.88 (0.75, 1.03)		0.89 (0.76, 1.05)	
Northeast	NA		1.03 (0.85, 1.25)		0.97 (0.80, 1.18)	
Southeast	NA		0.88 (0.73, 1.08)		0.84 (0.69, 1.03)	
*Tumor features*						
Stage						
Localized	NA		NA		Reference	<0.001
Regionalized	NA		NA		*2.86 (2.35, 3.49)*	
Distant	NA		NA		*16.05 (13.31, 19.37)*	
Unknown	NA		NA		*4.17 (3.15, 5.51)*	
Grade^b^						
Grade 1	NA		NA		Reference	<0.001
Grade 2	NA		NA		1.18 (0.94, 1.48)	
Grades 3 and 4	NA		NA		*1.83 (1.40, 2.39)*	
Unknown	NA		NA		*1.67 (1.29, 2.15)*	
Primary site						
Left	NA		NA		Reference	0.695
Right	NA		NA		1.02 (0.91, 1.15)	

*Note*: Italicized text indicates statistically significant result.

Abbreviations: CRC, colorectal cancer; HR, hazard ratio.

^a^ Others include divorced, separated, and widow.

^b^ Grade 1: well‐differentiated; Grade 2: moderately differentiated; Grade 3: poorly differentiated; Grade 4: undifferentiated.

^c^ Model 1 was adjusted for regional socioeconomic status; model 2 was adjusted for regional socioeconomic status and demographics; model 3 was adjusted for regional socioeconomic status, demographics, and tumor features.

## 4 DISCUSSION

We found that the 5‐year CRC survival rates were lower among Black patients in comparison to White patients (65.4% vs. 69.9%). This is consistent with prior literature that reported overall 5‐year CRC survival rates for Black adults have consistently been below that of White adults, with Black adults' overall survival rates at 61% and White adults' survival rates at 67% during 2000–2016 in the United States.^3^ Further, we found that Black patients living in low‐education (59.4% vs. 67.3%) regions appeared to have lower 5‐year survival rates compared to those not living in low‐education regions. Compared with findings from other studies, the 5‐year survival rate was 72.4% for the lowest SES quintile compared to 78.9% in the highest SES quintile for CRC patients,^23^ which is in line with our findings. Associations between neighborhood disadvantage and higher CRC stage at diagnosis and mortality have been found in other studies.^24^ For example, low neighborhood SES associated with lower CRC survival may be attributed to a lack of health care and preventative services access and utilization. Barriers to access to care include financial barriers due to high poverty, environmental barriers such as lack of transportation and a lack of nearby facilities, and social environment barriers such as cultural and linguistic factors, discrimination, and provider bias.^14^, ^15^, ^25^ Importantly, disparities in CRC screening contribute to the relatively high mortality from CRC among Blacks as compared with Whites. To impact these disparities, interventions should focus on both CRC screening utilizing high‐quality methods, and education on the value and accessibility of screening and appropriate treatment options for Black patients.^26^ CRC screening delivery approaches for underserved populations in particular should be provided in conjunction with programs and policies that provide access to follow‐up for diagnosis and appropriate treatment.^26^

In our multivariable analysis among White patients, we found that there were no statistically significant differences in CRC survival between patients living in persistent poverty and those not when adjusting for patient demographics only (Model 2) and without (Model 1). However, the significant impact of persistent poverty on CRC survival was found after adjusting for all covariates (Model 3). We found that White patients living in persistent poverty areas had a 12% increased risk of CRC death compared to those not living in persistent poverty. It is possible that tumor features, particularly for the stage at diagnosis, were a confounder that related to persistent poverty and CRC survival in our study. Consistent with a prior study, patients living in persistent poverty areas were more likely to have advanced diseases in CRC.^27^ CRC patients living in persistent poverty regions also had a 1.1‐fold increased risk of cancer‐specific mortality as compared with those without poverty.^27^ A possible explanation may be due to the limited availability of cancer screening, treatment, and care options for those patients living in persistent poverty, which resulted in a more advanced diagnosis and increased risk of CRC death.^8^, ^12^, ^13^

Unlike the results for White patients in our analysis, Black patients living in low‐education areas seem to have poorer CRC survival. We found that those living in regions with low education were 1.2–1.4‐fold more likely to die from CRC compared to those not living in this region regardless of covariates adjustment. Living in low SES regions, particularly in low education areas, was a more critical explanation for the higher risk of CRC death among Black patients in our study. Relative to Whites, Blacks tend to be diagnosed with CRC at younger ages and are more likely to be diagnosed with later‐stage cancer.^26^ However, racial differences in diagnostic outcomes were only found when Black patients were diagnosed with localized diseases in comparison to White patients (HR, 1.38; 95%CI, 1.11–1.71) in our analysis (Table S1). Black patients with localized CRC also demonstrated a lower 5‐year CRC survival than White patients (*p* = 0.008; Figure S1). More research is needed to further elucidate whether complex health status (e.g., multimorbidity) influences this finding. Because there are no studies that examined the relationship of area‐based education to CRC survival among non‐White patients, it is impossible to directly discuss our results with prior literature. Yet, a Finnish study using individual‐ and area‐level education to examine the impact of education on CRC survival found that educational inequalities due to geography in CRC survival were present only in women.^28^ Individual‐level education seems to be a stronger determinant of CRC survival, with a higher risk of CRC death found in the “basic education group” compared to the “high education group” in Finland.^28^ However, we were unable to compare results with individual‐level education due to unavailable information in the SEER program. Another study, using the area deprivation index (ADI) (education is one of the indicators), found that increasing ADI was associated with a 1.1‐fold increased risk of CRC mortality in Black patients.^17^ Therefore, these previous studies demonstrate that racial minorities and lower SES are more likely to experience poorer survival outcomes, which is consistent with our findings.

SES and racial disparities can in part explain CRC patient inequalities in access to early detection, suitable and appropriate quality care, and survival. Evidence has shown that lower neighborhood SES patients, particularly Black patients, were at significantly higher risk for delays and inadequate treatment for CRC.^29^, ^30^ In addition, Black patients' higher CRC mortality rates can be explained by unequal access to high‐quality surgical and oncology care, compared with White patients.^31^ Possible reasons for inadequate and delays in treatment may include fewer recommendations for treatment, multiple comorbidities, and patient refusal.^32^, ^33^ In addition, racial/ ethnic minorities residing in low SES regions may also experience limited availability of CRC screening for early detection. Particularly, colonoscopy is known to reduce CRC risk and mortality^34^; however, lower colonoscopy use was observed among Black individuals and those with low SES.^34^ Physicians whose patients are primarily minorities also tend to have less expertise and training in performing screening procedures and reduced access to clinical resources.^35^ Finally, systematic barriers in healthcare (e.g., financial barriers, lack of health insurance, racism) also prevent adequate cancer care and are associated with lower screening uptake, delays in diagnosis, decreased receipt of treatment, and lower treatment adherence.^36^ Together, these factors suggest the improvement of inequalities in access to high‐quality of cancer care and availability of CRC screening for racial minorities and low SES communities by appropriately allocating health resources may reduce the risk of CRC death in Georgia. Targeted CRC early detection should be prioritized for Black patients living in low‐education areas in Georgia. Health resources in early detection may include culturally tailored education programs developed specifically for high‐risk communities for CRC death, and increased access to healthcare facilities providing CRC screening in low‐resource areas or located far from the nearest resources.^30^

A major strength of this study is that we not only included patients' residential SES but also adjusted for individual‐level information regarding CRC diagnosis. Our findings may inform local health system policies for ongoing investments in communities with lower SES on CRC early detection and prevention in Georgia. For example, increasing the number of specialized healthcare professionals and CRC screening facilities to promote early detection and treatment in low‐resource communities. Other approaches, such as patient navigation programs, may help to ensure that patients newly diagnosed with CRC receive timely and appropriate treatment.^37^ Culturally tailored education programs through primary care and community initiatives are also needed to increase awareness of CRC risk and screening uptake, particularly for Black patients.^38^ Finally, more research should also examine whether the socio‐cultural differences impact CRC diagnosis outcomes in improving early CRC diagnosis and treatment among Black patients in Georgia.

Despite its strength, there were a few limitations that should be noted. Although the study has accounted for many covariates in analyses, the SEER database did not capture individual income and education level, lifestyle risk factors (e.g., obesity, smoking, and alcohol consumption), and concomitant diseases.^4^, ^24^, ^39^, ^40^ Particularly, SES is a complex matter combined with individual‐ and geographic‐area‐level influences. When possible, multiple indices of SES should be considered when analyzing relationships with health outcomes.^41^ The potential moderation and interaction between SES status at the individual‐, area level, and other factors (e.g., lifestyle risk factors and comorbidities) should also be considered. Second, barriers to cancer treatment and screening for CRC due to neighborhood socioeconomically disadvantaged backgrounds were also not available for investigation in this study. Factors, such as access to primary care practices and availability of CRC screening,^34^ can contribute to diagnosis outcomes. Third, we selected the higher burden regions of CRC mortality using Georgia public health districts, which may have the potential for including more specific race groups in the study; however, the overall racial composition in Georgia is 50% White and 33% Black with an increasing Hispanic/Latino population. Finally, although our study used traditional survival analyses which have been used in prior studies regarding analysis of CRC survival,^17^, ^39^ it may have potential bias due to non‐CRC or non‐cancer deaths. More research using alternative modeling approaches may further elucidate this relationship.

## 5 CONCLUSIONS

Racial differences in 5‐year CRC survival were observed in the five selected regions of Georgia, with significantly lower CRC survival rates in Black patients. The risk of CRC death was greater among White patients residing in regions with persistent poverty, and Black patients living in regions with low education. Findings from our study provide important evidence to all relevant stakeholders in allocating health resources (e.g., CRC screening and quality treatment options) aimed at CRC early detection and prevention by considering patients' regional SES in Georgia.

## AUTHOR CONTRIBUTIONS

**Meng‐Han Tsai:** Conceptualization (lead); data curation (lead); formal analysis (lead); funding acquisition (lead); methodology (equal); writing – original draft (lead); writing – review and editing (equal). **Marlo Vernon:** Conceptualization (equal); writing – review and editing (equal). **Shaoyong Su:** Methodology (equal); writing – review and editing (equal). **Steven S. Coughlin:** Writing – original draft (equal); writing – review and editing (equal). **Yanbin Dong:** Methodology (equal); supervision (equal); writing – review and editing (equal).

## FUNDING INFORMATION

This research was supported at least in part through the Georgia Cancer Center Paceline funding mechanism at Augusta University (principal investigator: Meng‐Han Tsai, MCGFD01050).

## CONFLICT OF INTEREST STATEMENT

The authors declare no potential conflict of interest.

## ETHICS STATEMENT

Data extracted for this study was publicly available and de‐identified and thus considered exempt from IRB review at Augusta University.

## Supporting information


Data S1.

## Data Availability

The datasets generated during the current study are available in the Surveillance, Epidemiology, and End Results Program (https://seer.cancer.gov/) and the US Department of Agriculture Economic Research Service (https://www.ers.usda.gov/data‐products/county‐typology‐codes/) repository.
