# Supplementary material for: Racial disparities in the relationship of regional socioeconomic status and colorectal cancer survival in the five regions of Georgia
Source: Cancer Med. 2024 Feb 13;13(3):e6954. doi: 10.1002/cam4.6954 (PMC10904969; doi:10.1002/cam4.6954)
Supplement: Supplementary file 1 — Data S1. [file CAM4-13-e6954-s001.docx]

**Supplementary Table 1. Association between race and CRC survival by stage at diagnosis**

|  | **Localized** ^a^ | | **Regionalized** ^a^ | | **Distant** ^a^ | |
| --- | --- | --- | --- | --- | --- | --- |
|  | **HR (95%CI)** | **P-value** | **HR (95%CI)** | **P-value** | **HR (95%CI)** | **P-value** |
| **Race** | | | | | | |
| White | Reference | 0.004 | Reference | 0.105 | Reference | 0.540 |
| Black | *1.38(1.11, 1.71)* |  | 1.12(0.98,1.28) |  | 1.03(0.92,1.14) |  |

Abbreviation: CRC, colorectal cancer; HR, hazard ratio. Italicized text indicates statistically significant result.

^a^ All models were adjusted for regional socioeconomic status, demographics, and tumor features. Other covariates are not shown. Results from unknown stage at diagnosis were also not shown.

| 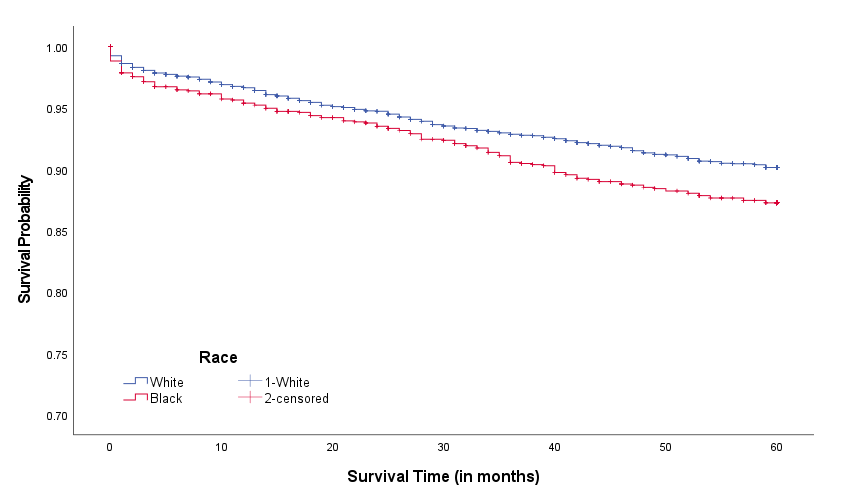 | 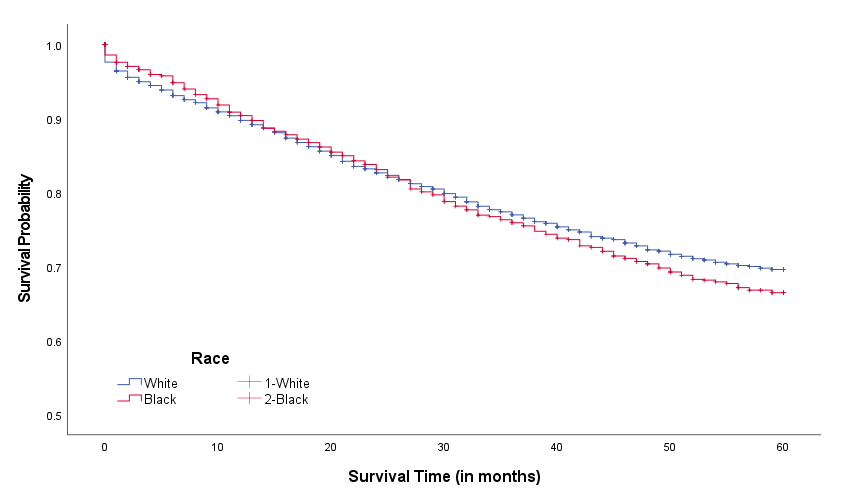 |
| --- | --- |
| **a) Localized colorectal cancer (p-value=0.008)** | **b) Regionalized colorectal cancer (p-value=0.157)** |
| 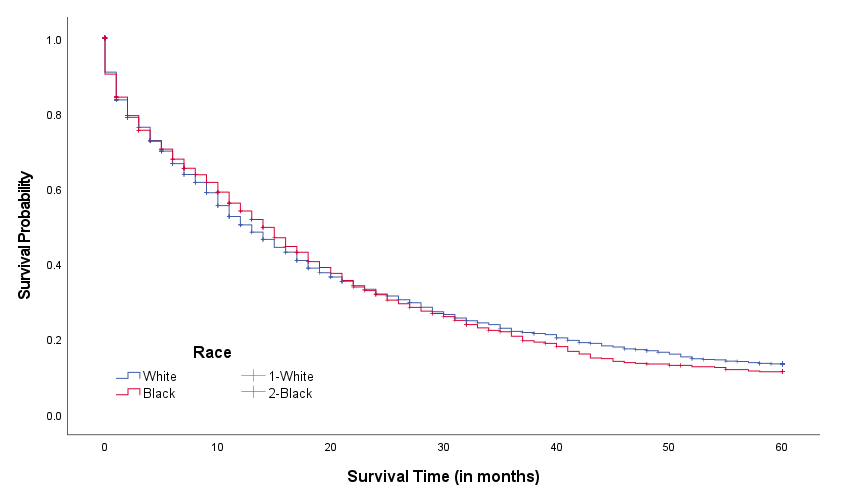 |  |
| **c) Distant colorectal cancer (p-value=0.704)** |  |
| **Supplementary Figure 1. Kaplan Meier colorectal cancer survival curves by stage at diagnosis.** Note: 1) Log-rank test was performed to examine survival rates. | |
